# β_1_-adrenergic receptor O-glycosylation regulates N-terminal cleavage and signaling responses in cardiomyocytes

**DOI:** 10.1038/s41598-017-06607-z

**Published:** 2017-08-11

**Authors:** Misun Park, Gopireddy R. Reddy, Gerd Wallukat, Yang K. Xiang, Susan F. Steinberg

**Affiliations:** 1 Department of Pharmacology, Columbia University, New York, NY USA; 2 Department of Pharmacology, University of California at Davis, Davis, CA USA; 3 Experimental and Clinical Research Center, Charité Campus Buch and Max-Delbrück Center for Molecular Medicine, Berlin, Germany; 4 VA Northern California Health Care System, Mather, CA USA

## Abstract

β_1_-adrenergic receptors (β_1_ARs) mediate catecholamine actions in cardiomyocytes by coupling to both Gs/cAMP-dependent and Gs-independent/growth-regulatory pathways. Structural studies of the β_1_AR define ligand-binding sites in the transmembrane helices and effector docking sites at the intracellular surface of the β_1_AR, but the extracellular N-terminus, which is a target for post-translational modifications, typically is ignored. This study identifies β_1_AR N-terminal O-glycosylation at Ser^37^/Ser^41^ as a mechanism that prevents β_1_AR N-terminal cleavage. We used an adenoviral overexpression strategy to show that both full-length/glycosylated β_1_ARs and N-terminally truncated glycosylation-defective β_1_ARs couple to cAMP and ERK-MAPK signaling pathways in cardiomyocytes. However, a glycosylation defect that results in N-terminal truncation stabilizes β_1_ARs in a conformation that is biased toward the cAMP pathway. The identification of O-glycosylation and N-terminal cleavage as novel structural determinants of β_1_AR responsiveness in cardiomyocytes could be exploited for therapeutic advantage.

## Introduction

β_1_-adrenergic receptors (β_1_ARs) are the principle mediators of catecholamine actions in cardiomyocytes. β_1_ARs rapidly adjust cardiac output by activating a Gs-adenylyl cyclase (AC) pathway that increases cAMP, activates protein kinase A (PKA), and phosphorylates substrates involved in excitation-contraction coupling. While β_1_ARs can also activate cardioprotective Gs-independent mechanisms via the recruitment of β-arrestin and transactivation of an epidermal growth factor receptor (EGFR) pathway that activates ERK^1^, chronic β_1_AR activation leads to a spectrum of changes (including cardiomyocyte hypertrophy/apoptosis, interstitial fibrosis, and contractile dysfunction) that contribute to the evolution of heart failure (HF)^2, 3^. βAR inhibitors that prevent maladaptive cAMP-driven βAR responses have become standard therapy for HF.

βARs have provided a useful prototype for structural and NMR spectroscopic studies designed to elucidate the molecular dynamics of G protein-coupled receptor (GPCR) activation^4–6^. However, studies to date have focused primarily on the human β_2_AR, the first hormone-activated GPCR to be cloned and structurally characterized^5, 6^. While β_1_- and β_2_ARs share considerable sequence homology in the transmembrane regions that form their ligand-binding pockets, other regions of these receptors are more divergent. In particular, the β_1_- and β_2_AR extracellular N-termini show no sequence homology. Since the βAR N-terminus has traditionally been viewed as having a negligible role in mechanisms that contribute to receptor activation and regulation, these relatively short and highly dynamic regions of the receptor generally are removed for structural studies. However, the notion that the βAR N-terminus can be dismissed as functionally unimportant is at odds with the fact that non-synonymous single nucleotide polymorphisms (SNPs) localized to the N-terminal regions of both the β_1_ and β_2_AR function as genetic determinants of βAR inhibitor responses and clinical outcome in HF^7^.

β_1_ and β_2_AR N-termini also are targets for sugar-based modifications (i.e., glycosylation). Protein glycosylation is an abundant post-translational modification that functions to expand the diversity of the proteome. Glycosylation is subdivided into two major categories (N- or O-glycosylation) based upon the residue within the protein backbone that serves as an attachment site for the branched sugar polymer (or glycan). N-glycosylation is initiated in the endoplasmic reticulum by the actions of an oligosaccharide transferase which catalyzes the *en bloc* transfer of a preformed complex glycan structure to the amide nitrogen on the side chain of an asparagine residue (in a Asn-x-Ser/Thr consensus sequence - where x is any residue other than proline^8^). The N-linked glycan structure then undergoes extensive modification during protein transport from the Golgi to the plasma membrane. In contrast, O-glycosylation is initiated by the transfer a single monosaccharide (generally α-GalNAc) to the hydroxyl group of an acceptor serine or threonine residue^8, 9^. This reaction (which is catalyzed by a polypeptide GalNAc-transferase, a multi-gene family of ~20 different enzymes) is then followed by the step-wise enzymatic transfer of additional sugars (including galactose, GlcNac, and fucose) to yield a spectrum of higher order linear and branched glycan structures. Both N- and O-linked glycan structures are then typically capped with negatively charged sialic acids. Glycan structures (in some cases on specific proteins) have been implicated in a vast number of key biological processes (including protein trafficking to membranes, cell adhesion, signal transduction, endocytosis) that are critical for normal embryonic development and normal organ physiology^8^. Recent studies also indicate that glycan structures are highly regulated during developmental and in response to environmental stimuli (conditions that leads to changes in the relative abundance and location of individual glycosyltransferase enzymes, the abundance and trafficking of glycoprotein substrates, and/or the availability of activated sugar donors) and that disordered protein glycosylation is a common feature of various inflammatory and metabolic disorders and a hallmark of certain cancers^10, 11^.

**Figure 1:**
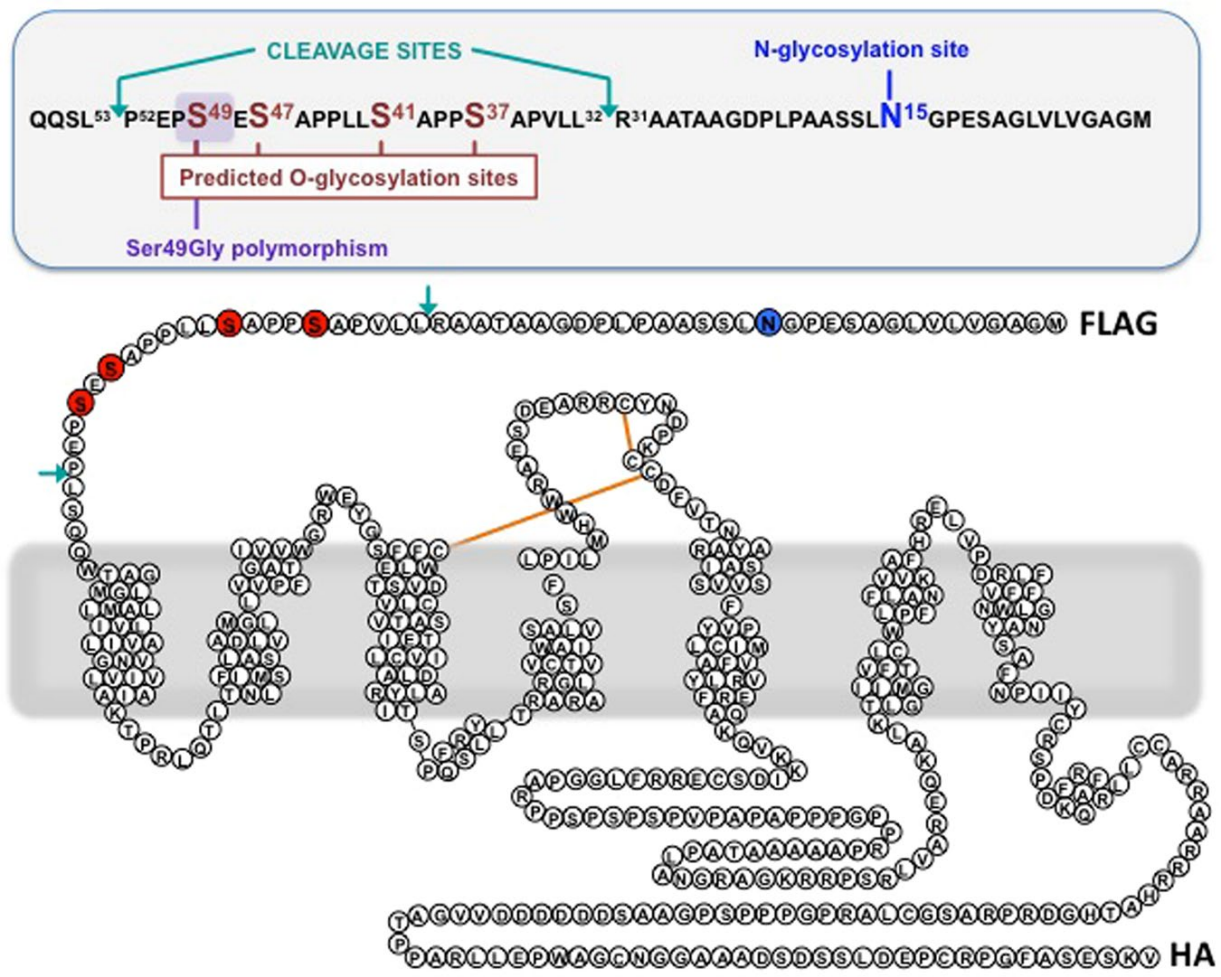
Schematic of the 2-dimensional topology of the human β_1_AR. The single N-glycosylation site in the N-terminus at position 15, in a consensus sequence [Nx(S/T)] is shown in blue. Putative O-glycosylation consensus sites (Ser residues in a Pro-rich environment) examined in this study are shown in red; the Ser49Gly polymorphism maps to a putative O-glycosylation site. Cleavage sites previously identified at the β_1_AR N-terminus are shown in turquoise^13^; cleavage at P52-L53 generates a non-glycosylated receptor. Constructs used for the experiments depicted in Figs 2, 3 and 6 contained N-terminal FLAG (DYKDDDDK) and C-terminal HA (YPYDVPDYA) tags.

βARs have both been categorized as glycoproteins, but the number and types of glycan attachments to the β_1_AR versus the β_2_AR are quite different. The β_2_AR contains 2 sites for N-linked glycosylation at its extreme N-terminus (at **N** ^6^GSAFLLAP**N** ^**15**^GS)^12^. β_2_AR N-glycosylation has been implicated as a mechanism that influences β_2_AR trafficking to cell surface membranes; it does not influence β_2_AR ligand binding or coupling to the Gs-cAMP pathway^12^. In contrast, the β_1_AR N-terminus contains a single site for N-linked glycosylation at Asn^15^ ^13^. β_1_AR N-glycosylation at this site is reported to influence β_1_AR homodimerization and β_1_AR heterodimerization with α_2_ARs; effects on ligand binding or agonist-induced internalization are not detected^14, 15^. However, the β_1_AR N-terminus also is the target for O-linked glycosylation^13^. Computational algorithms have been used to map this modification to a cluster of consensus O-glycosylation sites (i.e., Ser in a Pro-rich region) at S^37^, S^41^, S^47^, and S^49^ (Fig. 1)^13^, but the specific sites within the β_1_AR N-terminus that are targets for O-linked glycan modifications have never been unambiguously identified. This omission is pertinent, since Ser^37^, Ser^41^, and Ser^47^ are highly conserved in mammalian β_1_ARs, but sequence variation within the coding sequence of *ADRB1* gives rise to the Gly^49^ allele in ~20% of Caucasian and ~15% of African Americans^16^. The notion that a Ser49Gly polymorphism might alter β_1_AR O-glycosylation patterns and thereby underlie this human SNP’s function as a clinically important modifier of βAR inhibitor responsiveness and outcome in patients with HF^16^ has never been considered. Similarly, the consequences of β_1_ARs O-glycosylation remain uncertain. There is evidence that clusters of O-glycans in other membrane-bound proteins alter protein secondary structure, serve as ligands for cell adhesion, and can be sources of antigen for the immune system^17^. Mucin-like O-glycans also have been implicated as barriers that prevent protein cleavage by proteases^17^, a property that might be particularly relevant the β_1_AR since the human β_1_AR (like its turkey counterpart) undergoes N-terminal cleavage at sites adjacent to the putative O-glycosylation sites (Fig. 1 and refs 13 and 18). This study uses molecular and biochemical strategies to map O-glycosylation sites on the β_1_AR N-terminus and show that O-glycosylation regulates β_1_AR cleavage, a process that in turn influences β_1_AR signaling properties in cardiomyocytes.

## Results

### β_1_AR Glycosylation Sites

β_1_AR glycosylation patterns were interrogated in Chinese hamster ovary (CHO) *ldlD* cells, a cell line that is UDP-galactose/UDP-N-acetylgalactosamine 4-epimerase-deficient. *ldlD* cells cannot synthesize UDP-Gal or UDP-GalNAc, the nucleotide sugars required for the addition of galactose (Gal) and N-acetylgalactosamine (GalNAc) to N- or O-linked oligosaccharides on glycoproteins, under conventional culture conditions with glucose as the sole sugar source^19^. However, the 4-epimerase defect can be bypassed and oligosaccharide synthesis can be fully restored by the addition of Gal and GalNAc to the culture medium, making *ldlD* cells a unique resource to map β_1_AR glycosylation sites.

**Figure 2:**
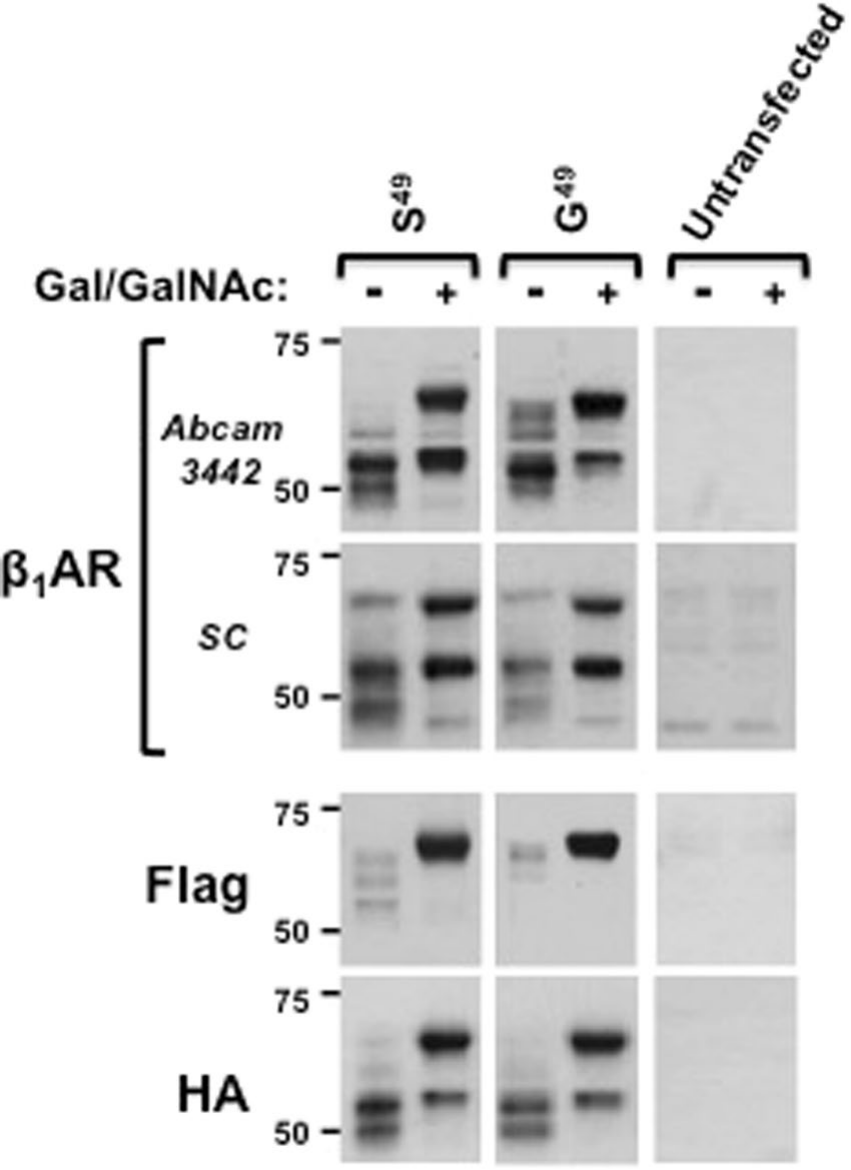
The S49G polymorphic variant does not grossly influence β_1_AR glycosylation profiles in *ldlD* cells. Lysates from *ldlD* cells transiently transfected with plasmids that drive expression of either S^49^- or G^49^-β_1_ARs and cultured without or with Gal (20 μM) and GalNAc (200 μM) were subjected to immunoblot analysis with two different anti-β_1_AR antibodies as well as antibodies to Flag and HA epitope tags at the N- and C-termini, respectively. The figure shows that the Abcam anti-β_1_AR antibody (ab3442, raised against residues 394–408 in human β_1_ARs) and the Santa Cruz anti-β_1_AR antibody (raised against some undefined C-terminal epitope) both detect the heterologously overexpressed β_1_AR. However, immunoblots with the Santa Cruz anti-β_1_AR antibody also contain some non-specific immunoreactivity. In particular, a ~69-kDa non-specific band that co-migrates with the full-length, fully glycosylated β_1_AR can become rather problematic at low levels of transgene expression. Therefore, the Abcam reagent (which is more sensitive and specific) was used in subsequent experiments.

Initial studies examined the expression patterns for WT-Ser^49^-β_1_ARs and WT-Gly^49^-β_1_ARs (the two β_1_AR polymorphic variants, see Fig. 1) in *ldlD* cells grown without or with Gal/GalNAc. Figure 2 shows that in the absence of Gal/GalNAc, both β_1_AR variants accumulate primarily as cleaved ~48–52-kDa species that are recognized by anti-β_1_AR and anti-HA (antibodies directed against C-terminal epitopes), but not by anti-Flag (which recognizes the N-terminal epitope tag). The very small amounts of a somewhat larger β_1_AR species that is produced under these conditions (and is detected by both anti-β_1_AR and anti-Flag) is presumed to represent a partially glycosylated species that is produced as a result of a low level of sugar scavenging from serum glycoproteins^20^. Addition of Gal/GalNAc to the culture medium leads to the accumulation of two larger β_1_AR species; a full-length ~69-kDa β_1_AR that retains both N-terminal Flag and C-terminal HA tags and a smaller truncated form of the receptor that lacks the N-terminal Flag tag. The migration of the ~69-kDa full-length β_1_AR is considerably slower than predicted from the calculated molecular weight of a full-length unglycosylated epitope-tagged receptor (~55-kDa) suggesting that the protein contains N- and/or O-linked glycans. Studies with Ser^49^- and Gly^49^-β_1_AR variants yielded similar results, indicating that β_1_AR glycosylation patterns are not grossly influenced by the S49G polymorphism. It is important to note that anti-β_1_AR antibodies from two different commercial sources (Abcam 3442 and Santa Cruz sc-568) replicated the results with anti-HA; anti-β_1_AR and anti-HA antibodies detected bands with identical mobilities in transfected (but not non-transfected) cells under all experimental conditions. These results effectively address recent concerns regarding the specificity of the antibodies used to detect GPCRs such as the βAR^21, 22^ and indicate that these bands represent *bona fide* transgene products.

The observation that β_1_ARs accumulate in *ldlD* cells as full-length proteins only in the presence of Gal/GalNAc indicates that glycosylation in some way prevents β_1_AR cleavage. This could suggest a role for N- or O-linked sugars on the β_1_AR itself, but a mechanism involving other cellular glycoconjugates is not excluded. Therefore, we mapped β_1_AR glycosylation sites and determined whether β_1_ARs glycosylation prevents β_1_AR cleavage.

**Figure 3:**
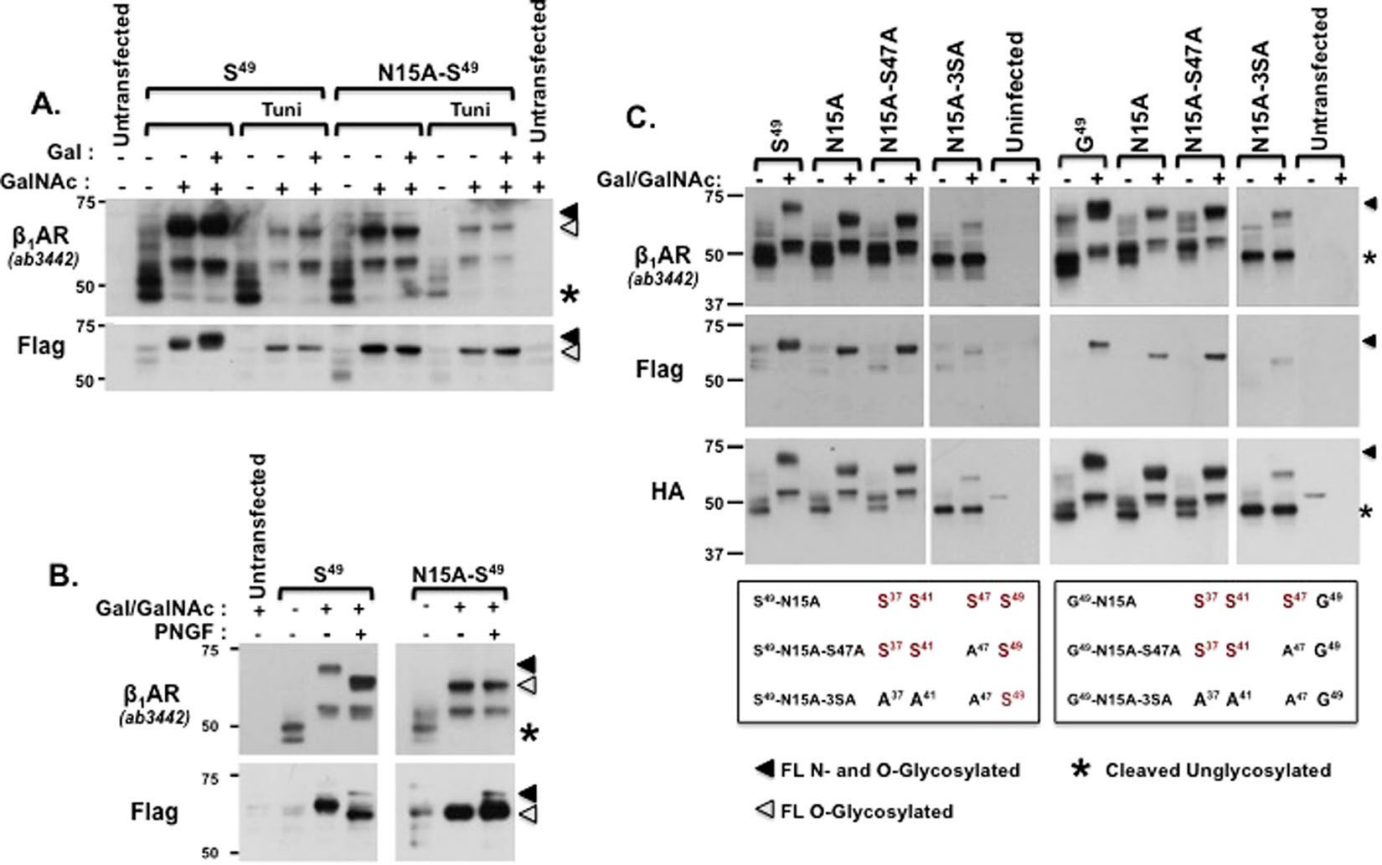
Mutagenesis studies to map β_1_AR N-terminal glycosylation sites. *ldlD* cells transfected with wild type or single residue substituted forms of the β_1_AR were cultured without or with Gal (20 μM) and GalNAc (200 μM) as indicated. Tunicamycin (Tuni) was added to the culture medium (1 μg/ml for 24 hr) to block N-glycosylation in *Panel A*. Samples were incubated with PNGase F (PNGF 2 U/mL, 16 hr) to hydrolyze N-linked sugars in *Panel B*. Lysates were subjected to immunoblot analysis with an anti-β_1_AR antibody (raised against a β_1_AR C-tail epitope) or antibodies to Flag and HA epitope tags at the N- and C-termini, respectively. Positions of the full-length fully N- and O-glycosylated β_1_AR (filled triangle), the full-length O-glycosylated receptor (that lacks the N^15^-linked glycan, open triangle) and the N-terminally-cleaved unglycosylated receptors (asterisk) are indicated.

We first used biochemical and mutagenesis approaches to identify β_1_AR species that contain N-linked glycans. Figures 3A and B show that the mobility of the major ~69-kDa species recognized by the anti-β_1_AR and anti-Flag antibodies increases when β_1_ARs are synthesized in the presence of tunicamycin (which prevents N-linked chain additions) or treated with PNGase F (which specifically hydrolyzes N-linked sugars); these results indicate that the ~69-kDa band contains N-linked glycans. The smaller ~55-kDa band detected by the anti-β_1_AR antibody (but not anti-Flag) is not influenced by tunicamycin or PNGF, indicating that this is an N-terminally cleaved species and that cleavage occurs C-terminal to the N-glycosylation site. Figure 3 shows that N15A-β_1_AR is detected as N-terminally truncated ~48- and 52-kDa species in *ldlD* cells cultured without Gal/GalNAc and that β_1_AR-N15A accumulates as two larger species in the presence of Gal/GalNAc: [1] a full length ~66-kDa species that is recognized by both anti-β_1_AR and anti-Flag antibodies - that has an electrophoretic mobility identical to WT-β_1_AR treated with tunicamycin or PNGF - and [2] a truncated ~55-kDa species that is selectively recognized by anti-β_1_AR, but not anti-Flag. β_1_AR-N15A is not influenced by tunicamycin or PNGF treatment. These results indicate that β_1_ARs contain a single site for N-glycosylation at position 15, that N-glycosylation is not required for full length β_1_AR expression, and that N-glycosylation does not grossly regulate β_1_AR N-terminal cleavage.

The observation that a N15A substitution prevents β_1_AR N-glycosylation, but does not discernibly alter β_1_AR processing/maturation provided the rationale to use the N-glycosylation-defective N15A-β_1_AR construct as a backbone for subsequent studies designed to identify sites for O-glycosylation. We focused on a cluster of consensus O-glycosylation sites at the juxtamembrane region of β_1_AR N-terminus, introducing S-A substitutions at positions 37, 41, and 47 into either Ser^49^-N15A-β_1_AR or Gly^49^-N15A-β_1_AR backbones.

Figure 3C shows that the introduction of a single S47A substitution into either in the Ser^49^-N15A-β_1_AR or Gly^49^-N15A-β_1_AR background does not alter the relative abundance or the mobility of the β_1_AR species that accumulate in *ldlD* cells cultured without or with Gal/GalNAc. These results effectively exclude Ser^47^ and Ser^49^ as a β_1_AR O-glycosylation sites or sites that regulate β_1_AR cleavage. Rather, Fig. 3C shows that β_1_AR mutants that harbor additional S-A substitutions at positions 37 and 41 (the Ser^49^-N15A-3SA-β_1_AR or Gly^49^-N15A-3SA-β_1_AR constructs) accumulate as an N-terminally cleaved ~48-kDa species (detected by anti-β_1_AR and anti-HA, but not anti-Flag) in *ldlD* cells incubation either without or with Gal/GalNAc. These results effectively map β_1_AR O-glycosylation sites to Ser^37^ and/or Ser^41^ and implicate O-glycosylation at these sites as a modification that prevents β_1_AR N-terminal cleavage.

**Figure 4:**
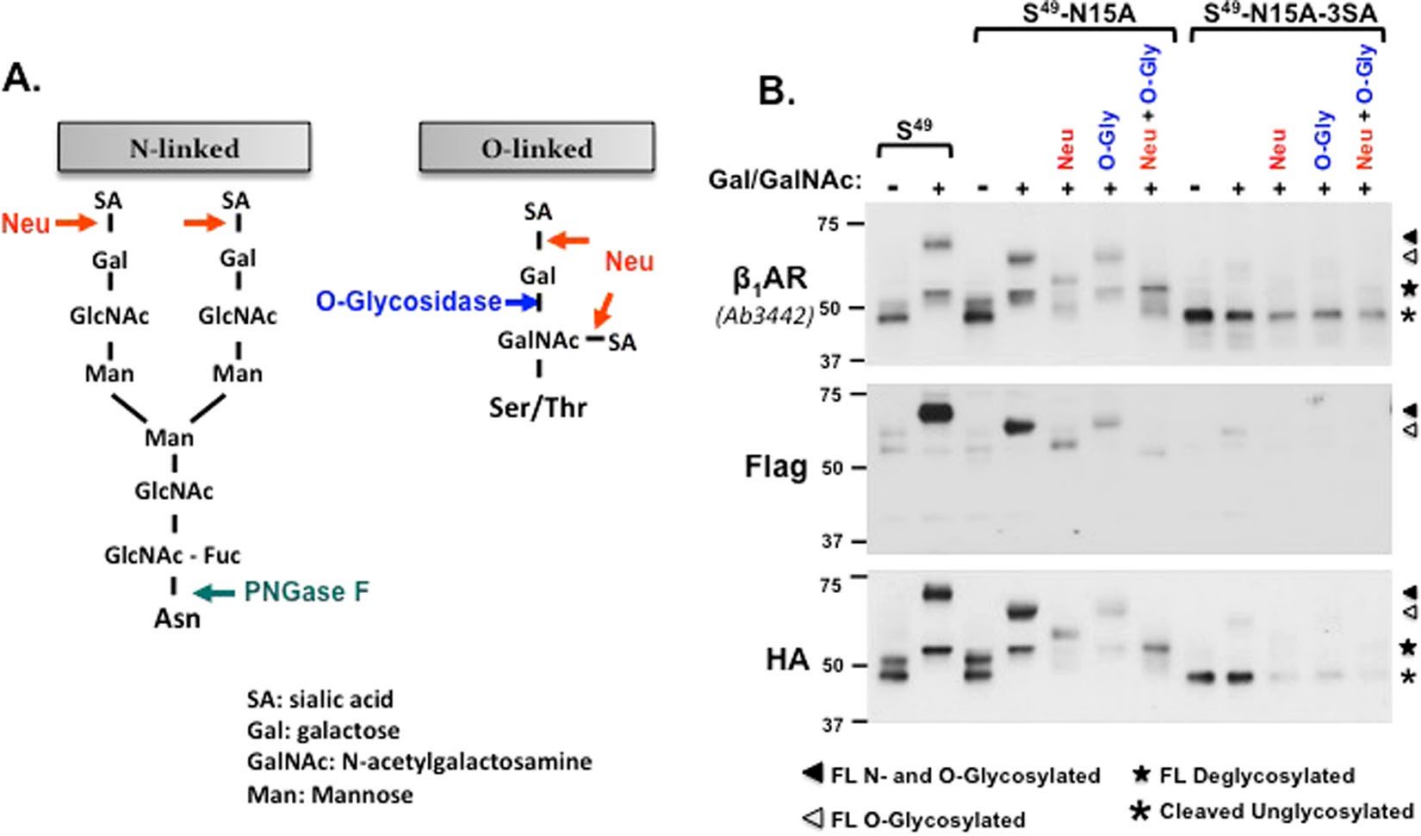
β_1_ARs carry sialylated N- and O-linked glycans that are released by enzymatic deglycosylation. *Panel A*: Schematics showing cleavage sites in representative N- or O-linked glycan structures. Enzymatic deglycosylations were performed with Neuraminidase (Neu), O-Glycosidase (O-Gly), and Peptide-N-glycosidase F (PNGase F). *Panel B:* Lysates from *ldlD* cells transfected with S^49^-β_1_AR, S^49^-β_1_AR-N15A, or S^49^-β_1_AR-N15A-3SA cultured without or with Gal (20 μM) and GalNAc (200 μM) were subjected to deglycosylation protocols as described in Methods. Lysates were then probed with antibodies that recognize C-terminal epitopes (anti-β_1_AR and anti-HA) or N-terminal Flag. Positions of the full-length fully N- and O-glycosylated β_1_AR (filled triangle), the full-length O-glycosylated receptor (that lacks the N^15^-linked glycan, open triangle), the full-length deglycosylated receptor (star), and the N-terminally-cleaved unglycosylated receptors (asterisk) are indicated.

O-glycosylation was assessed further with neuraminidase (which removes terminal sialic acids from N- and O-linked sugars) and O-glycosidase (which removes O-linked glycan cores from glycoproteins only after the terminal sialic acid has been removed by neuraminidase). Figure 4 shows that neuraminidase treatment increases the electrophoretic mobility of N15A-β_1_ARs. Since the N15A substitution prevents β_1_AR N-glycosylation, these results indicate that β_1_ARs contain sialylated O-linked glycans. Treatment with O-glycosidase alone does not alter N15A-β_1_AR mobility, but the full-length ~65-kDa Ser^49^-N15A-β_1_AR species collapses to a ~55-kDa species following combined treatment with O-glycosidase + neuraminidase. Deglycosylation experiments performed in parallel on N15A-3SA-β_1_ARs and showed that the N15A-3SA-β_1_AR species that accumulates in Gal/GalNAc-treated *ldlD* cells is not influenced by O-glycosidase and/or neuraminidase treatments (i.e., this species is neither sialylated nor O-glycosylated). Deglycosylation experiments on the Gly^49^ variant of the β_1_AR harboring N15A or N15A-3SA-β_1_AR yielded identical results (data not shown). Collectively, these results indicate that β_1_ARs accumulate as O-glycosylated species in *ldlD* cells cultured with Gal/GalNAc, that O-glycosylation sites also are heavily sialylated, and that O-linked sugar modifications at Ser^37^ and/or Ser^41^ prevent β_1_AR cleavage.

Finally, these experimental protocols were replicated using an untagged β_1_AR construct to address the possible concern that the epitope tags might influence β_1_AR maturation/glycosylation. These additional studies were considered important since a C-terminal tag on the β_2_AR subtype (which like the β_1_AR, terminates in a S-*x*-*ϕ* class I PDZ binding motif) disrupts PDZ domain-mediated protein interactions and prevents β_2_AR recycling to surface membranes following agonist-induced internalization^23^; N-terminal tags on βAR subtypes typically are viewed as functionally silent. Figure S1A shows that an untagged β_1_AR construct is detected as a ~69-kDa band, corresponding to the full-length β_1_AR, in *ldlD* cells grown with (but not without) Gal/GalNAc. Figure S1B shows that the electrophoretic mobility of this ~69-kDa band increases in response to treatment with PNGase F (indicating that it contains N-linked glycans) and that neuraminidase and O-glycosidase treatments produce further increases in this band’s electrophoretic mobility over that produced by PNGase F treatment alone (indicating that it also contains sialylated O-linked glycans). The observation that the untagged β_1_AR is processed (like the tagged β_1_AR) to a ~69-kDa protein that contains sialylated N- and O-linked glycans in *ldlD* cells grown with Gal/GalNAc establishes that the N- and C-terminal tags do not influence β_1_AR glycosylation.

### β_1_ARs accumulate as both full-length and N-terminally cleaved species in cardiomyocytes

**Figure 5:**
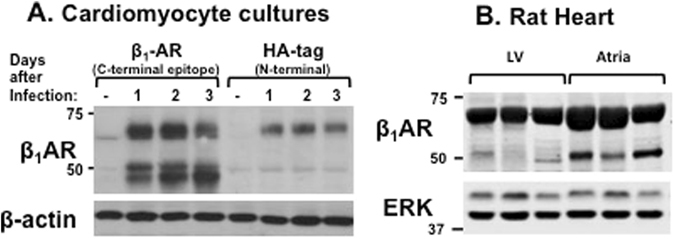
β_1_AR are detected as full-length and truncated species in cardiomyocytes. *Panel A:* Adenoviral-mediated gene delivery was used to overexpress N-terminally tagged β_1_ARs in neonatal rat cardiomyocyte cultures. Lysates were prepared at 1–3 days following infection and probed for β_1_ARs, with β-actin used as a protein loading control. *Panel B:* Lysates prepared from adult left ventricular (LV) or atrial tissues were probed for β_1_AR expression (with ERK protein serving as loading control, since the anti-β-actin antibody - which gives a very robust signal in various cell culture preparations - performed poorly in lysates prepared from intact rat hearts). A shorter exposure of the β_1_AR immunoblot is included as Fig. S2.

We previously identified distinct molecular forms of the β_1_AR in rat cardiomyocyte cultures and rat ventricle and concluded that these distinct molecular species represent *bona fide* β_1_AR gene products, since similar molecular heterogeneity of β_1_ARs is detected in the hearts of wild type mice but not β_1_AR null mutants^24, 25^. However, the previous immunoblotting studies relied exclusively on antibodies directed against C-terminal epitopes and could not resolve β_1_AR mobility differences due to receptor cleavage versus other mechanisms (for example, differential glycosylation or oxidative modifications at extracellular loop cysteines that influence intramolecular disulfide bond formation)^26^. Therefore, adenoviral-mediated gene transfer was used to overexpress N-terminally HA-tagged β_1_ARs in cardiomyocytes. Figure 5A shows that an antibody directed against a β_1_AR C-terminal epitope detects the transgene as both a larger ~69-kDa and two smaller ~45–50-kDa species and that only the larger ~69-kDa species carries the N-terminal HA-tag. These results indicate that the smaller species is the product of N-terminal cleavage in cardiomyocytes. Figure 5A also shows that both full-length and truncated forms of the β_1_AR are detected within 24 hr of adenoviral infection and their levels remain stable for up to 3 days.

Recent studies identify atrial-ventricular differences in glycosylation-associated gene (glycogene) expression that lead to chamber-specific differences in protein glycosylation^27^. Therefore, we performed immunoblotting as a general screen to identify tissue-specific difference in β_1_AR processing. Figure 5B shows that β_1_ARs are detected as a ~69-kDa species in membranes prepared from rat left ventricle and atrium and that atrium is relatively enriched in a smaller ~52-kDa species that co-migrates with the N-terminally truncated β_1_AR.

### N-terminal truncation alters β_1_AR signaling in cardiomyocytes

We generated an N-terminally truncated form of the β_1_AR based upon a cleavage site identified by N-terminal sequencing in a previous study^13^ and packaged this construct into an adenoviral vector to drive β_1_AR expression in cardiomyocytes. Untagged β_1_AR constructs were packaged into adenoviral vectors for these expression studies, to avoid any possible confounding effects of the epitope tags. Preliminary radioligand binding experiments established that membranes with similar levels of either full length (FL) or N-terminally truncated β_1_AR (Δ2-52-β_1_AR) immunoreactivity contain similar numbers of [^125^I]CYP binding sites (838 ± 91 *vs*. 725 ± 62 fmol/mg; n = 4, NS) that bind [^125^I]CYP with similar affinity (65.5 ± 29.4 *vs*. 77.5 ± 15.7 pM; n = 4, NS). The observation that an N-terminal truncation does not impair β_1_AR interactions with an antagonist ligand is consistent with structural studies that map the β_1_AR ligand binding pocket to residues in transmembrane helices and the second extracellular loop^28^.

**Figure 6:**
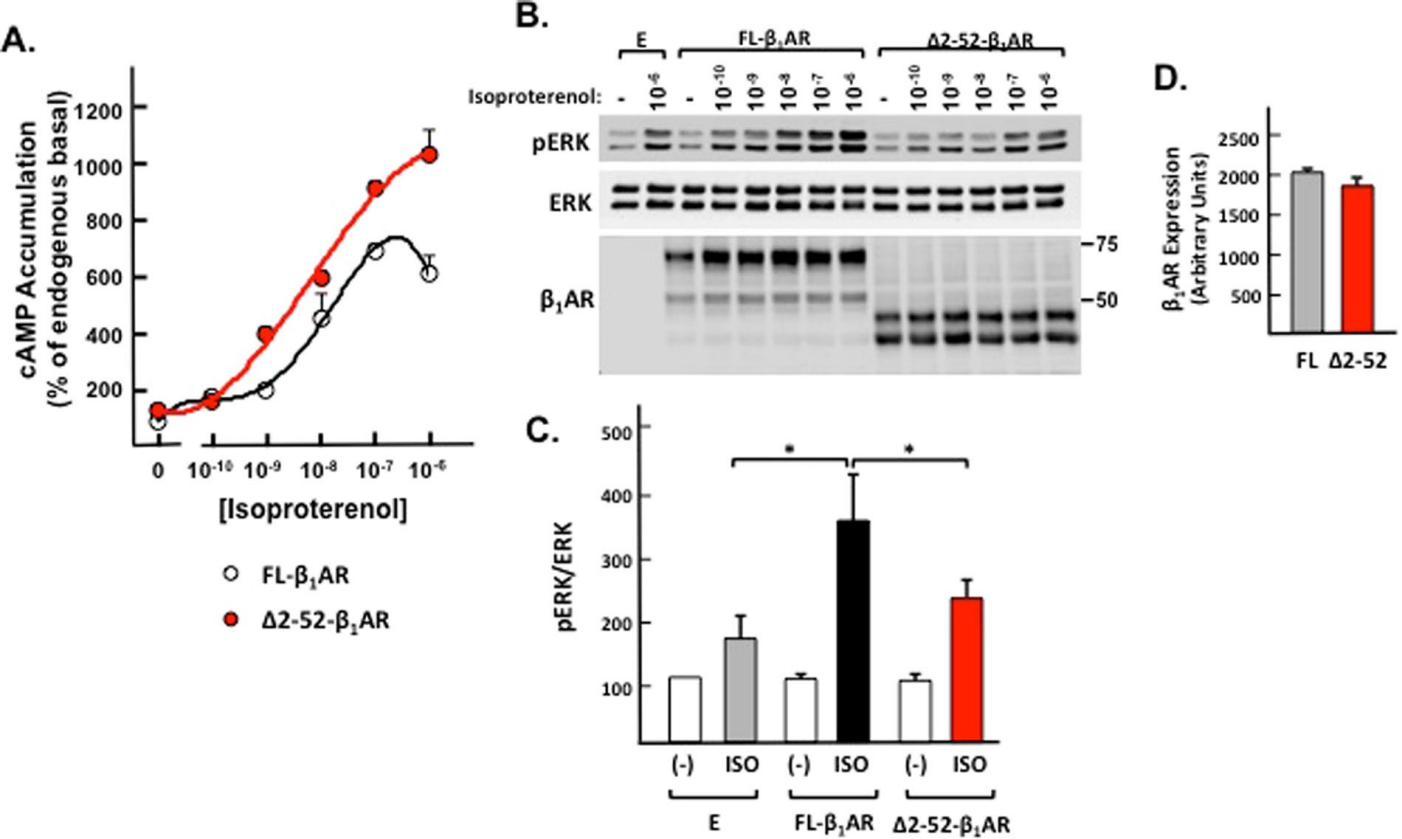
N-terminal truncation influences β_1_AR signaling to cAMP versus ERK pathways in cardiomyocytes. Neonatal cardiomyocyte cultures were infected with empty vector (E) or adenoviruses that drive expression of FL (Ad-FL-β_1_AR) or truncated (Ad-∆2-52-β_1_AR) forms of the β_1_AR. *Panel A*: Cultures were preincubated with 10 mM theophylline for 1 h at 37 °C and then challenged with vehicle or a range of isoproterenol concentrations and cAMP accumulation was measured according to Methods. Analysis by ANOVA followed by Tukey’s test showed that maximal cAMP accumulation is higher in Ad-∆2-52-β_1_AR than in Ad-FL-β_1_AR cultures (p < 0.05, n = 3). *Panel B:* Lysates from cultures treated for 5 min with vehicle or a range of isoproterenol concentrations were subjected to immunoblot analysis for ERK protein and phosphorylation and β_1_AR protein expression. *Panel C:* Quantification of pERK (normalized to ERK protein) in resting and 10^−6^ M isoproterenol-treated cultures infected with empty vector, FL-β_1_AR or ∆2-52-β_1_AR showing that basal pERK/ERK ratios are not influenced by Ad-FL-β_1_AR or Ad-∆2-52-β_1_AR overexpression, but Isoproterenol-dependent ERK phosphorylation is higher in Ad-FL-β_1_AR than in Ad-∆2-52-β_1_AR cultures (mean ± SEM, n = 6, *p < 0.05). *Panel D:* FL-β_1_AR and ∆2-52-β_1_AR expression was quantified (by combining signals from the two immunoreactive species detected for each construct) and did not differ (mean ± SEM, n = 6, NS).

Cardiomyocytes that heterologously overexpress similar levels of FL or N-terminally truncated β_1_ARs were challenged with a range of isoproterenol concentrations to determine whether N-terminal cleavage alters β_1_AR signaling to Gs/cAMP versus ERK pathways. Figure 6 shows that Ad-Δ2-52-β_1_AR cultures display a higher level of cAMP accumulation (and reduced ERK phosphorylation) in response to a range of isoproterenol concentrations, compared to Ad-FL-β_1_AR cultures. While a previous study identified higher levels of the cleaved β_1_AR species following long-term (6 hr) agonist activation (and concluded that β_1_ARs are cleaved upon agonist activation^13^), the more short term isoproterenol treatment used in this study does not alter the abundance of any β_1_AR species in cardiomyocytes. These results argue that the N-terminal cleavage that is detected in cardiomyocytes is a stable modification that is completed prior to the delivery of the β_1_AR to the cell surface membrane and is not dynamically regulated by agonist activation. Rather, our studies indicate that N-terminal cleavage functions to alter the balance of β_1_AR signaling to the Gs/cAMP versus the ERK signaling pathway.

### β_1_AR glycosylation influences responses to agonistic autoantibodies

**Figure 7:**
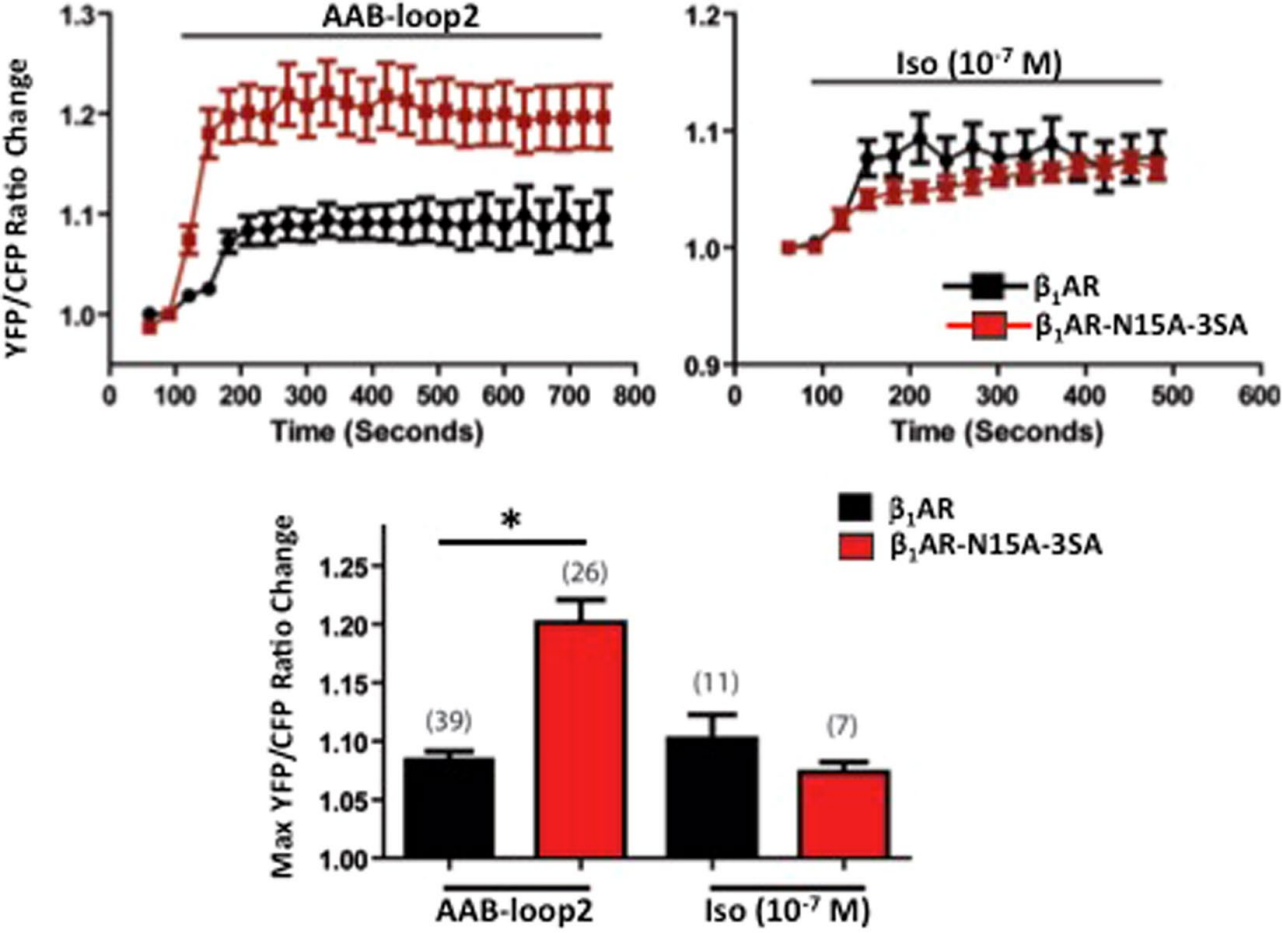
β_1_AR O-glycosylation influences responses to agonistic autoantibodies. Neonatal rat cardiomyocytes that heterologously overexpress PM-AKAR3 (the plasma membrane-localized PKA activity FRET biosensor) plus similar amounts of either S^49^-WT-β_1_AR or glycosylation defective S^49^-β_1_AR-N15A-3SA were stimulated with β_1_AR agonistic autoantibodies (AAB-loop2) or isoproterenol (10^−7^ M) and FRET was measured at 30 sec intervals. Note: The S^49^-WT-β_1_AR and S^49^-β_1_AR-N15A-3SA constructs used in these experiments are identical to the constructs used for the biochemical studies in Figs 2–4. *Top:* Time course for agonist-induced changes in FRET ratios (normalized to resting levels before drug). *Bottom:* Maximum FRET responses to AABs and Iso. *p < 0.01 by one-way ANOVA followed by Tukey’s test.

Autoantibodies against the 2^nd^ extracellular loop of the β_1_AR accumulate in certain heart failure syndromes (Chagas’ disease, dilated cardiomyopathy, ischemic cardiomyopathy) and contribute to the pathogenesis of these disorders by binding and activating the β_1_AR. We examined whether post-translational processing events localized to the β_1_AR N-terminus influence the β_1_AR responsiveness to anti-β_1_AR agonistic autoantibodies (AABs). The studies took advantage of membrane-targeted A-Kinase Activity Reporter (AKAR), a FRET reporter that senses plasma membrane localized PKA activity^29^ (a localized signal that is regulated by a local pool of cAMP and may not necessarily track the global change in cAMP accumulation detected in Fig. 6). Figure 7 shows that both isoproterenol and anti-β_1_AR AABs increase plasma membrane PKA activity in cells that express WT-β_1_AR and glycosylation-defective β_1_AR-N15A-3SA. While the isoproterenol-dependent increase in membrane PKA activity is more robust at early time points (2–4 min) in WT-β_1_AR cells compared to β_1_AR-N15A-3SA cells, this difference wanes with more prolonged incubations. However, at all time points, the glycosylation-defective β_1_AR-N15A-3SA elicits a markedly exaggerated response to agonistic anti-β_1_AR AABs compared to WT-β_1_ARs. It’s worth noting that the AABs were better than isoproterenol at stabilizing β_1_AR-N15A-3SA in a conformation that activates membrane-localized PKA; a similar superior efficacy of some AABs batches - compared to isoproterenol - has been reported by others (although the notion that AABs might be particularly efficacious at only certain molecular forms of the β_1_ARs was not previously considered)^30^. These results support the conclusion that β_1_ARs are stabilized in a conformation that is biased toward the cAMP pathway as a result of defective O-glycosylation and/or the resultant N-terminal truncation.

## Discussion

Protein O-glycosylation is an evolutionarily conserved mechanism that can enhance the functional diversity of a target protein. Most studies have focused on the dense clusters of GalNAc-type O-glycan modifications that decorate mucin-domains of relatively abundant secreted proteins that can be subjected to large-scale purification for analytic methods. However, recent studies suggest that GalNAc-type O-glycosylation is a more common post-translational modification that also occurs at isolated sites on a wide range of proteins without mucin-like features. Progress toward identifying these other O-glycosylation sites (and in particular, the identification of O-glycosylation sites on cell surface proteins, such as G protein-coupled receptors) and efforts to understand the functional significance of O-glycan modifications in normal development and/or the pathogenesis of various clinical disorders has lagged considerably at least in part due to several formidable technical challenges: [1] O-linked glycan structures are non-template driven modifications that characteristically shows high levels of structural diversity (‘microheterogeneity’), even at a single site within a given protein. [2] Pharmacologic compounds that specifically inhibit polypeptide GalNAc transferases or enzymes that can be used to quantitatively release all O-glycan structures from protein backbones are not available. [3] The necessary and sufficient elements that comprise a consensus O-glycosylation motif remain uncertain; bioinformatics algorithms have been trained on a selective set of known *O*-glycoproteins and do not predict most sites identified in the human O-GalNAc glycoproteome using newly developed mass spectrometry methods^31^. [4] Available analytic techniques remain cumbersome and have substantial limitations. While there are isolated reports that O-glycan modifications decorate the N-termini of certain G protein-coupled receptors (V2 vasopressin, LDL, and chemokine receptors^32–34^) and that O-linked sugars on the LDL receptor itself (rather than on other cellular components) prevent receptor N-terminal proteolytic cleavage and stabilize LDL receptors on the cell surface^33^, most studies of βARs have focused on the N-linked glycan modifications that can be detected on both β_1_AR and β_2_ARs. The O-glycan modifications that are confined to β_1_ARs are seldom considered. This study maps β_1_AR O-glycosylation sites and implicates O-glycosylation as a mechanism that prevents β_1_AR N-terminal cleavage and influences β_1_AR signaling responses in cardiomyocytes.

We used a mutagenesis approach to show that β_1_AR O-glycosylation is confined to Ser^37^/Ser^41^ and does not involve Ser^49^, the site of a clinical relevant SNP^35^. This result is seemingly at odds with a previous report from the Liggett laboratory, which identified distinct electrophoretic mobilities for Ser^49^- and Gly^49^-β_1_ARs stably overexpressed in clonal CHW lines and speculated that this difference reflects a indirect/long-range effect of the position 49 polymorphism to alter β_1_AR N-glycosylation at Asn^15^; a role for Ser^49^ O-glycosylation was not considered^36^. In fact, we obtained these clonal cell lines from the Liggett laboratory and replicated their findings; we believe that the distinct migration patterns for Ser^49^- and Gly^49^-β_1_ARs in the clonal cell lines generated in the Liggett laboratory can be attributed to the presence of some inopportune genetic alteration (at the level of the β_1_AR itself or some other protein that influences β_1_AR maturation/post-translational processing) in one of their clonal cells lines, since the electrophoretic mobilities of Ser^49^- and Gly^49^-β_1_ARs stably overexpressed in clonal lines generated in our laboratory or transiently overexpressed in a range of other cell types do not differ. Rather, our studies effectively exclude a role for Ser^49^ as a direct substrate for O-linked glycosylation or an indirect regulator of N-glycosylation.

We used a number of strategies to examine the functional consequences of β_1_AR O-glycosylation. First, we showed that O-glycosylation is required for full-length β_1_AR expression; β_1_ARs accumulate as N-terminally truncated forms as a result of the O-glycosylation defect that results from Gal/GalNAc deprivation in *ldlD* cells or site-directed mutagenesis. There is precedent for this type of interplay between O-glycosylation and proteolytic cleavage, with site-specific O-glycosylation at juxtamembrane regions of certain other membrane proteins (in some cases regulated by specific ppGalNAc-T isoforms with tissue-restricted patterns of expression) conferring structural stability and protecting against proteolytic cleavage (i.e., preventing ectodomain shedding)^37^. The identification of an O-glycosylation/N-terminal cleavage mechanism that gives rise to distinct molecular forms of the β_1_AR provides the first credible explanation for the molecular heterogeneity displayed by native β_1_ARs in various cardiac preparations; the endogenous β_1_AR in cardiomyocyte cultures and mouse ventricle is detected as two distinct species, a larger ~69-kDa band (corresponding to the full-length/glycosylated receptor) and a smaller ~50-kDa band (that co-migrates with the N-terminally truncated β_1_AR).

An O-glycosylation regulated event that regulates β_1_AR processing is predicted to have important functional implications since the cardiac glycome is extensively remodeled during normal ventricular developmental and in the setting of cardiac hypertrophy^27, 38, 39^. It is interesting to speculate that developmental- or disease-associated changes in the relative abundance of ppGalNAcT family enzymes that initiate O-glycosylation), the repertoire of glycosyltransferase enzymes that extend the core O-glycan structure (that produce diverse ensembles of branched glycan structures), or the expression of sialyltransferase enzymes that cap O- and N- linked glycans with sialic acid might impact on β_1_AR glycosylation (and secondarily β_1_AR cleavage and β_1_AR responsiveness). The observation that glycan-modifying enzyme expression and protein glycosylation patterns are regulated in a tissue-specific manner (including between atrial *versus* ventricular tissues^27^) provides a likely explanation for the atrial-ventricular difference in the abundance of the smaller molecular form of the β_1_AR that co-migrates with N-terminally truncated β_1_ARs. Collectively, these results provide a strong rationale to consider functionally-important glycosylation-driven changes in β_1_AR structure as a dynamically regulated mechanism that contributes to the pathogenesis of various cardiac phenotypes. However, an analysis of site-specific glycan modifications on native β_1_ARs in various cardiac tissues (to characterize the structural changes in O-glycan moieties that accompany or contribute to the pathogenesis of cardiac diseases) remains technically challenging even with the most contemporary O-glycoproteomic methods (see ref 40). More sophisticated analytic strategies that can be used to dissect glycan structural diversity, particularly on native O-glycoproteins in complex biological samples, are under development and will be critical for future progress in this area.

Our studies identify an O-glycosylation-regulated N-terminal cleavage event as a mechanism that alters β_1_AR signaling bias to cAMP/PKA versus ERK pathways. With the caveat that the functional studies were performed in overexpression models (and ultimately must be followed-up by studies that interrogate glycosylation/cleavage mechanisms that control the expression and action of native β_1_ARs at endogenous levels of β_1_AR expression), these studies suggest that an O-glycosylation-regulated mechanism that dictates β_1_AR responsiveness could underlie the cardiac phenotypes that develop in various syndromes associated with defective in glycoprotein glycosylation. For example, congenital disorders of glycosylation due to mutations or deletion of genes that encode the enzymes that form the core O-glycan structures typically present with lethal ventricular arrhythmias and cardiomyopathies^41^. The molecular basis for this glycosylation-driven cardiac phenotype remains uncertain. Studies to date have linked defects in protein glycosylation (that disrupt protein sialylation) to changes in the gating properties of certain voltage-gated sodium and potassium channels, enhanced cardiac excitability, and increased susceptibility to ventricular arrhythmias^27, 42–44^. While changes in ion channel sialylation may contribute to the pathogenesis of the ventricular arrhythmias in these disorders, our studies provide a rationale to consider whether defective β_1_AR O-glycosylation and the accumulation of an N-terminally truncated β_1_AR species that displays enhanced signaling to proarrhythmic cAMP/PKA responses also might contribute to this pathologic cardiac phenotype.

Ventricular arrhythmias also are a characteristic feature of acquired disorders of protein sialylation, such as Chagas disease. *Trypanosoma cruzi* (the causative agent of Chagas disease) releases a trans-sialidase that transfers sialic acid from glycoconjugates on the host cell to mucin-like proteins on the parasite cell surface. This results in changes in immune cell sialylation that effectively subvert some aspects of the host cell immune response^45, 46^. Of note, early studies linked *Trypanosoma cruzi* infection to changes in βAR responsiveness that in some cases correlate with the severity of chagasic cardiomyopathy^47–49^. The prevailing notion is that the disordered immune response leads to the generation of agonistic anti-β_1_AR autoantibodies that activate the cAMP signaling pathway and contribute to chagasic cardiomyopathy. While there is direct evidence that agonistic anti-β_1_AR autoantibody treatment results in a cardiomyopathic phenotype^50^, the limited correlation between circulating levels of anti-β_1_AR autoantibodies and the severity of cardiac dysfunction^51^ suggests that other mechanisms also may be contributory. Our studies provide the rationale to consider whether a *Trypanosoma cruzi* infection induced decrease in β_1_AR sialylation might facilitate β_1_AR N-terminal cleavage, enhance agonistic antibody-dependent activation of the cAMP/PKA pathway (while dampening signaling via the cardioprotective ERK pathway), and promote adverse cardiac remodeling.

Finally, our studies identify the β_1_AR extracellular N-terminus as a heretofore unrecognized structural determinant of β_1_AR responsiveness. The notion that a glycan-regulated event localized to the N-terminus can influence β_1_AR responsiveness represents a paradigm shift from previous research that focused almost exclusively on the ligand binding sites in transmembrane helices or effector docking sites in the intracellular loops and the C-terminus. However, our results resonate with a small but growing literature that link structural perturbations (and/or changes in glycosylation) localized to the N-terminus to altered GPCR cell surface expression or compartmentation to lipid raft microdomains, changes in the kinetics of ligand-induced internalization, changes in the efficiency of receptor dimerization, and altered signaling bias to downstream effectors^14, 52, 53^. The identification of an O-glycan-regulated cleavage event that regulates β_1_AR signaling to cAMP/PKA vs ERK provides a strong rational for future studies that identify the O-glycan modifying enzymes and specific protease(s) that execute these post-translational modifications at the β_1_AR N-terminus and the specific role of glycan-mediated changes in β_1_AR structure/function in the pathogenesis of various cardiac disorders. The identification of O-glycosylation and/or receptor cleavage sites that are specific to the β_1_AR N-terminus, that add plasticity to catecholamine-dependent signaling responses, could represent promising novel targets for β_1_-subtype specific therapeutics.  

### **Note**

While this manuscript was under revision, a publication from Goth et al. described the sites and functional consequences of beta1-adrenergic receptor O-glycosylation (JBC 292:4714,2017).  We believe that methodologic differences explain certain discrepancies between the findings in this recent publication and the results reported herein. First, Goth et al. used in vitro O-glycosylation assays with peptides based upon the β1-adrenergic receptor N-terminus and recombinantly expressed GalNAc-transferase 2 to identify O-glycosylation at Ser37/Ser41 and Ser47/Ser49.  Our studies, which show that Ser47and Ser49 are not O-glycosylated in vivo in GalNAc-transferase 2-expressing ldlD-CHO cells, suggest that the in vitro approach is too promiscuous to be used as a surrogate to predict in vivo O-glycosylation sites on the full length β1-adrenergic receptor protein.  Second, Goth et al. linked an O-glycosylation defect to a decrease in Iso-dependent cAMP accumulation. However, this conclusion was based on studies in β1-adrenergic receptor-expressing GalNac-T2/T3 knock-out HEK293 cells and may be misleading, since this cell model has a generalized defect in O-glycosylation of β1-adrenergic receptors, adenylyl cyclase, and a wide array of other cellular proteins. Our studies show that the truncated/glycosylation-defective β1-adrenergic receptor couples to enhanced cAMP accumulation in cardiomyocytes (a physiologically relevant cell type).

## Materials and Methods

### Materials

Antibodies were from the following sources: anti-β_1_AR (clone V-19, which was raised against a peptide that maps to the C-terminus of the mouse β_1_AR) and anti-HA (clone Y-11) were from Santa Cruz Biotechnology (Dallas, TX). Anti-β_1_AR (ab3442, raised against residues 394–408 in human β_1_-ARs) and anti-β-actin were from Abcam (Cambridge, MA). Anti-Flag M2 antibody was from Sigma-Aldrich (Saint Louis, MO). Antibodies that recognize ERK protein and phosphorylation were from Cell Signaling Technology (Danvers, MA). Goat anti-rabbit and goat anti-mouse IgG (H + L)-Horse radish peroxidase conjugates were from Bio-Rad Laboratories, Inc. (Hercules, CA). Peptide-N-glycosidase F (PNGF), O-glycosidase (O-Gly), neuraminidase (Neu), propranolol, isoproterenol (Iso), and tunicamycin were obtained from Sigma-Aldrich (Saint Louis, MO). All other chemicals were reagent grade.

### Plasmids

A plasmid that drives expression of the human S^49^R^389^-β_1_AR harboring an N-terminal Flag-tag and C-terminal HA-tag was from Addgene. The various single residue substituted β_1_AR mutant constructs used in this study were generated using the QuikChange mutagenesis system (Agilent Technologies). A plasmid that drives expression of the N-terminally truncated human β_1_AR (GenBank^TM^ accession number P08588) (∆2-52-β_1_AR) harboring a C-terminal Flag tag was kindly provided by Dr. Ulla E. Petäjä-Repo from University of Oulu, Finland^54^. Adenoviruses that drive expression of the full-length and N-terminally truncated forms of the human β_1_AR (Ad-FL-β_1_AR and Ad-∆2-52-β_1_AR) were prepared by Welgen Inc. (Worcester, MA).

### HEK293 and ldlD cell culture and transfection

HEK293 cells were cultured in DMEM (Gibco life technologies) containing 10% FBS, 100 units/ml penicillin-streptomycin, and 2mM L-glutamine. *ldlD* cells were cultured in DMEM/F-12 (Ham) (1:1) (Gibco life technologies) with 10% FBS and 100 units/ml penicillin-streptomycin either without or with Gal (20 μM) and GalNAc (200 μM). Cell transfections were performed with the Effectene Transfection reagent (Qiagen) according to manufacturer’s instructions.

### Neonatal Cardiomyocyte Culture and Adenoviral Infections

Cardiomyocytes were isolated from the hearts of 2-day-old Wistar rats and infected with adenoviral constructs that drive expression of full length or N-terminally truncated β_1_ARs according to methods published previously^55, 56^. All procedures were performed in accordance with the *Guide for the Care and Use of Laboratory Animals published by the US National Institutes of Health* (NIH Publication, 8th Edition, 2011) and were approved by the Columbia University Institutional Animal Care and Use Committee (protocol AC-AAAH6903).

### Immunoblotting

Immunoblotting was performed on cell extracts according to methods described previously or manufacturer’s instructions^55^. The dilutions for primary and secondary antibodies were as follows: Abcam anti-β_1_AR (ab3442) at 1:3000 followed by secondary goat anti-rabbit IgG at 1:5000; Santa Cruz anti-β_1_AR (clone V-19) at 1:1000 followed by secondary goat anti-rabbit IgG at 1:2000; anti-HA (Y-11) at 1:3000 followed by secondary goat anti-rabbit IgG at 1:5000; anti-Flag M2 at 1:700 followed by secondary goat anti-mouse IgG at 1:1000; anti-pERK at 1:2000 followed by secondary goat anti-rabbit IgG at 1:3000; anti-ERK at 1:3000 followed by secondary goat anti-rabbit IgG at 1:5000; anti-β-actin at 1:2500 followed by secondary goat anti-mouse IgG at 1:4000. Each panel in each figure represents the results from a single gel (exposed for a uniform duration); detection was with enhanced chemiluminescence or LI-COR Odyssey CLx imaging system (LI-COR Biosciences) with image Studio Lite Ver 5.0 software used for quantification of protein expression. All results were replicated in at least three experiments on separate culture preparations.

### Enzymatic deglycosylation

Samples were deglycosylated by preincubation with peptide-N-glycosidase F (PNGase F), O-glycosidase (O-Gly), α-(2 → 3, 6, 8, 9)-neuraminidase (Sialidase A, Neu) for overnight at 37 °C using Enzymatic Protein Deglycosylation Kit (EDEGLY kit, Sigma) according to manufacturer’s instructions.

### Measurements of βAR affinity and cAMP accumulation

Radioligand binding experiments with [^125^I]ICYP were performed on membrane preparations according to methods published previously^57^. cAMP accumulation was measured according to standard methods as described previously^58^. In brief, cells cultured in 6-well plates, infected with adenoviral constructs, and then 3 days later preincubated with 10 mM theophylline for 60 min and challenged for 5 min with vehicle or Iso. Assays were terminated by aspiration of the incubation buffer and addition of 0.5 mL of 100% ice-cold ethanol to each well. Cell lysates were dried in a spin vacuum and cAMP in the residue was quantified with a commercially available cAMP enzyme-linked immunosorbent assay kit (R&D Systems, Minneapolis, MN) according to manufacturer’s instructions.

### Fluorescent Resonance Energy Transfer (FRET) Measurements to track sarcolemmal PKA activity

Neonatal cardiomyocyte cultures were infected with PM-AKAR3 (a plasma membrane-targeted PKA activity reporter) according to methods described previously^29^. Images were acquired using a Leica DMI3000B inverted fluorescence microscope (Leica Biosystems, Buffalo Grove, IL) with a 40X oil-emersion objective lens and a charge-coupled device camera controlled by Metafluor software (Molecular Devices, Sunnyvale, CA). FRET was recorded by exciting the donor fluorophore at 430–455 nm and measuring emission fluorescence with two filters (475DF40 for cyan and 535DF25 for yellow). Images were subjected to background subtraction, and were acquired every 20 seconds with exposure time of 200 ms. The donor/acceptor FRET ratio was calculated and normalized to the ratio value of baseline. The binding of cAMP to AKAR3 increases YFP/CFP FRET ratio^59^.

### Statistics

Results are shown as mean ± SEM and were analyzed by Student’s *t* test or ANOVA for multiple comparisons, with P < 0.05 considered statistically significant.

## Electronic supplementary material

Supplementary Information

## References

[1] Noma T (2007). β-Arrestin-mediated β_1_-adrenergic receptor transactivation of the EGFR confers cardioprotection. J Clin Invest.

[2] Port JD, Bristow MR (2001). Altered β-adrenergic receptor gene regulation and signaling in chronic heart failure. J Mol Cell Cardiol.

[3] Lohse MJ, Engelhardt S, Eschenhagen T (2003). What is the role of β-adrenergic signaling in heart failure?. Circ Res.

[4] Bokoch MP (2010). Ligand-specific regulation of the extracellular surface of a G-protein-coupled receptor. Nature.

[5] Rosenbaum DM (2007). GPCR engineering yields high-resolution structural insights into β_2_-adrenergic receptor function. Science.

[6] Rasmussen SG (2007). Crystal structure of the human β_2_-adrenergic G-protein-coupled receptor. Nature.

[7] Dorn GW, Liggett SB (2008). Pharmacogenomics of β-adrenergic receptors and their accessory signaling proteins in heart failure. Clin Transl Sci.

[8] Stanley P (2016). What have we learned from glycosyltransferase knockouts in mice?. J Mol Biol.

[9] Tian E, Ten Hagen KG (2009). Recent insights into the biological roles of mucin-type O-glycosylation. Glycoconj J.

[10] Tran DT, Ten Hagen KG (2013). Mucin-type O-glycosylation during development. J Biol Chem.

[11] Kailemia MJ, Park D, Lebrilla CB (2017). Glycans and glycoproteins as specific biomarkers for cancer. Anal Bioanal Chem.

[12] Rands E (1990). Mutational analysis of β-adrenergic receptor glycosylation. J Biol Chem.

[13] Hakalahti AE (2010). Human β_1_-adrenergic receptor Is subject to constitutive and regulated N-terminal cleavage. J Biol Chem.

[14] He J, Xu J, Castleberry AM, Lau AG, Hall RA (2002). Glycosylation of β_1_-adrenergic receptors regulates receptor surface expression and dimerization. Biochem Biophys Res Commun..

[15] Xu J (2003). Heterodimerization of α_2A_ and β_1_-adrenergic receptors. J Biol Chem.

[16] Liggett SB (2010). Pharmacogenomics of β_1_-adrenergic receptor polymorphisms in heart failure. Heart Fail.Clin..

[17] Hang HC, Bertozzi CR (2005). The chemistry and biology of mucin-type O-linked glycosylation. Bioorg Med Chem.

[18] Jurss R, Hekman M, Helmreich EJ (1985). Proteolysis-associated deglycosylation of β_1_-adrenergic receptor in turkey erythrocytes and membranes. Biochemistry.

[19] Kingsley DM, Kozarsky KF, Hobbie L, Krieger M (1986). Reversible defects in O-linked glycosylation and LDL receptor expression in a UDP-Gal/UDP-GalNAc 4-epimerase deficient mutant. Cell.

[20] Kozarsky KF, Call SM, Dower SK, Krieger M (1988). Abnormal intracellular sorting of O-linked carbohydrate-deficient interleukin-2 receptors. Mol Cell Biol.

[21] Hamdani N, van der Velden J (2009). Lack of specificity of antibodies directed against human β-adrenergic receptors. Naunyn-Schmiedeberg’s Arch Pharmacol.

[22] Kirkpatrick P (2009). Specificity concerns with antibodies for receptor mapping. Nat Rev Drug Discov.

[23] Cao TT, Deacon HW, Reczek D, Bretscher A, von Zastrow M (1999). A kinase-regulated PDZ-domain interaction controls endocytic sorting of the β_2_-adrenergic receptor. Nature.

[24] Rybin VO, Xu X, Lisanti MP, Steinberg SF (2000). Differential targeting of β-adrenergic receptor subtypes and adenylyl cyclase to cardiomyocyte caveolae. J Biol Chem.

[25] Rohrer DK (1996). Targeted disruption of the mouse β_1_-adrenergic receptor gene: developmental and cardiovascular effects. Proc Natl Acad Sci USA.

[26] Moxham CP, Ross EM, George ST, Malbon CC (1988). β-adrenergic receptors display intramolecular disulfide bridges *in situ*: analysis by immunoblotting and functional reconstitution. Mol Pharm.

[27] Montpetit ML (2009). Regulated and aberrant glycosylation modulate cardiac electrical signaling. Proc Natl Acad Sci USA.

[28] Warne T, Edwards PC, Leslie AG, Tate CG (2012). Crystal structures of a stabilized β_1_-adrenoceptor bound to the biased agonists bucindolol and carvedilol. Structure.

[29] Liu S (2012). Phosphodiesterases coordinate cAMP propagation induced by two stimulatory G protein-coupled receptors in hearts. Proc Natl Acad Sci USA.

[30] Bornholz B (2013). Impact of human autoantibodies on β_1_-adrenergic receptor conformation, activity, and internalization. Cardiovasc Res.

[31] Steentoft C (2013). Precision mapping of the human O-GalNAc glycoproteome through SimpleCell technology. EMBO J.

[32] Sadeghi H, Birnbaumer M (1999). O-Glycosylation of the V2 vasopressin receptor. Glycobiology.

[33] Kozarsky K, Kingsley D, Krieger M (1988). Use of a mutant cell line to study the kinetics and function of O-linked glycosylation of low density lipoprotein receptors. Proc Natl Acad Sci USA.

[34] Szpakowska M (2012). Function, diversity and therapeutic potential of the N-terminal domain of human chemokine receptors. Biochem Pharmacol.

[35] Dorn GW, Liggett SB (2009). Mechanisms of pharmacogenomic effects of genetic variation within the cardiac adrenergic network in heart failure. Mol Pharmacol.

[36] Rathz DA, Brown KM, Kramer LA, Liggett SB (2002). Amino acid 49 polymorphisms of the human β_1_-adrenergic receptor affect agonist-promoted trafficking. J Cardiovasc Pharm.

[37] Goth CK (2015). A systematic study of modulation of ADAM-mediated ectodomain shedding by site-specific O-glycosylation. Proc Natl Acad Sci USA.

[38] Rong J (2014). Glycan imaging in intact rat hearts and glycoproteomic analysis reveal the upregulation of sialylation during cardiac hypertrophy. J Am Chem Soc.

[39] Yang S (2015). Integrated glycoprotein immobilization method for glycopeptide and glycan analysis of cardiac hypertrophy. Anal Chem.

[40] Levery SB (2015). Advances in mass spectrometry driven O-glycoproteomics. Biochim Biophys Acta.

[41] Hennet T, Cabalzar J (2015). Congenital disorders of glycosylation: a concise chart of glycocalyx dysfunction. Trends Biochem Sci.

[42] Schwetz TA, Norring SA, Ednie AR, Bennett ES (2011). Sialic acids attached to O-glycans modulate voltage-gated potassium channel gating. J Biol Chem.

[43] Ednie AR, Horton KK, Wu J, Bennett ES (2013). Expression of the sialyltransferase, ST3Gal4, impacts cardiac voltage-gated sodium channel activity, refractory period and ventricular conduction. J Mol Cell Card.

[44] Ufret-Vincenty CA (2001). Role of sodium channel deglycosylation in the genesis of cardiac arrhythmias in heart failure. J Biol Chem.

[45] Freire-de-Lima L (2010). Trypanosoma cruzi subverts host cell sialylation and may compromise antigen-specific CD8+ T cell responses. J Biol Chem.

[46] Freire-de-Lima L, Fonseca LM, Oeltmann T, Mendonca-Previato L, Previato JO (2015). The trans-sialidase, the major Trypanosoma cruzi virulence factor: Three decades of studies. Glycobiology.

[47] Morris SA (1991). Myocardial β-adrenergic adenylate cyclase complex in a canine model of chagasic cardiomyopathy. Circ Res.

[48] Morris SA, Tanowitz HB, Rowin KS, Wittner M, Bilezikian JP (1984). Alteration of the pattern of β-adrenergic desensitization in cultured L6E9 muscle cells infected with Trypanosoma cruzi. Mol Biochem Parasitol.

[49] Nussinovitch U, Shoenfeld Y (2013). The clinical significance of anti-β_1_-adrenergic receptor autoantibodies in cardiac disease. Clin Rev Allergy Immunol.

[50] Jahns R (2004). Direct evidence for a β_1_-adrenergic receptor-directed autoimmune attack as a cause of idiopathic dilated cardiomyopathy. J Clin Invest.

[51] Talvani A (2006). Levels of anti-M2 and anti-β_1_ autoantibodies do not correlate with the degree of heart dysfunction in Chagas’ heart disease. Microbes Infect.

[52] Marada S (2015). Functional divergence in the role of N-linked glycosylation in smoothened signaling. PLoS Genet.

[53] Cho DI (2012). The N-terminal region of the dopamine D2 receptor, a rhodopsin-like GPCR, regulates correct integration into the plasma membrane and endocytic routes. Br J Pharmacol.

[54] Hakalahti AE (2013). β-adrenergic agonists mediate enhancement of β_1_-adrenergic receptor N-terminal cleavage and stabilization *in vivo* and *in vitro*. Mol Pharmacol.

[55] Ozgen N (2008). Protein kinase D links Gq-coupled receptors to cAMP response element-binding protein (CREB)-Ser^133^ phosphorylation in the heart. J Biol Chem.

[56] Steinberg SF, Robinson RB, Lieberman HB, Stern DM, Rosen MR (1991). Thrombin modulates phosphoinositide metabolism, cytosolic calcium, and impulse initiation in the heart. Circ Res.

[57] Steinberg SF, Zhang H, Pak E, Pagnotta G, Boyden PA (1995). Characteristics of the β-adrenergic receptor complex in the epicardial border zone of the 5-day infarcted canine heart. Circulation.

[58] Steinberg SF, Robinson RB, Lieberman HB, Stern DM, Rosen MR (1991). Thrombin modulates phosphoinositide metabolism, cytosolic calcium, and impulse initiation in the heart. Circ Res.

[59] Soto D, De Arcangelis V, Zhang J, Xiang Y (2009). Dynamic protein kinase A activities induced by β-adrenoceptors dictate signaling propagation for substrate phosphorylation and myocyte contraction. Circ Res.

