# Supplementary material for: β1-adrenergic receptor O-glycosylation regulates N-terminal cleavage and signaling responses in cardiomyocytes
Source: Sci Rep. 2017 Aug 11;7:7890. doi: 10.1038/s41598-017-06607-z (PMC5554155; doi:10.1038/s41598-017-06607-z)
Supplement: Supplementary file 1 — Supplementary Information [file 41598_2017_6607_MOESM1_ESM.pdf]

β_1-adrenergic receptor O-glycosylation regulates N-terminal cleavage and signaling responses in cardiomyocytes

Misun Park, Gopireddy R Reddy, Gerd Wallukat, Yang K. Xiang, and
Susan F. Steinberg

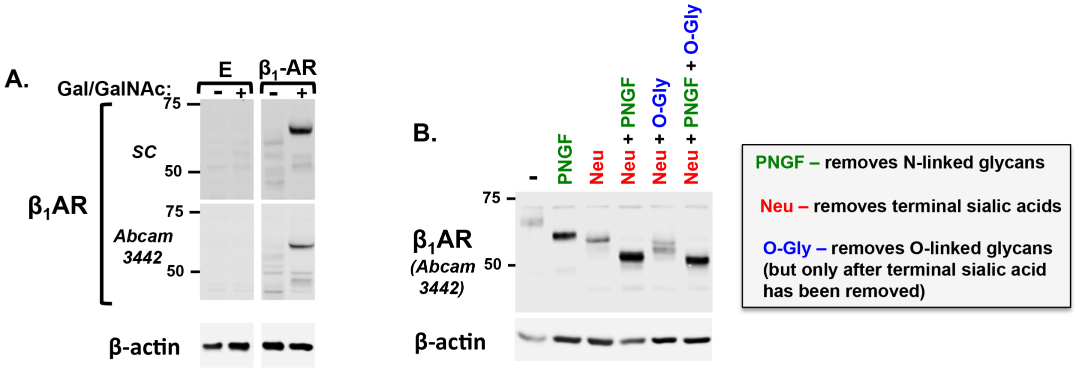

Figure S1. Glycosylation profile of an untagged β_1AR. *Panel A:* An untagged β_1AR was heterologously overexpressed in *ldlD* cells cultured without or with Gal (20 μM) and GalNAc (200 μM) as indicated. Immunoblot analysis with two different β_1AR antibodies shows that a ~69-kDa β_1AR species (that corresponds to the full-length fully glycosylated β_1AR) and lesser amounts of smaller β_1AR species accumulate only when ldlD cells are cultured with Gal/GalNAc. *Panel B*: The β_1AR species that accumulate in ldlD cells cultured with Gal/GalNAc were subjected to deglycosylation protocols. The deglycosylation experiment establishes that that the ~69-kDa band contains N-linked glycans (since its mobility increases in response to treatment with PNGF) as well as sialylated O-linked glycans (since neurominidase and O-glycosidase produce further increases in β_1AR electrophoretic mobility - over than produced by PNGF treatment alone). These results indicate that epitope tags on the N- and C-terminus do not grossly influence maturational processing (glycosylation) of the β_1AR.

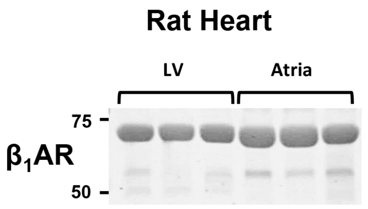

Figure S2. Lighter exposure of Figure 5B.
